# Insect Models in Nutrition Research

**DOI:** 10.3390/biom12111668

**Published:** 2022-11-11

**Authors:** Miray Tonk-Rügen, Andreas Vilcinskas, Anika E. Wagner

**Affiliations:** 1 Institute of Nutritional Science, Justus Liebig University, Wilhelmstrasse 20, 35392 Giessen, Germany; 2 Institute for Insect Biotechnology, Justus Liebig University, Heinrich-Buff-Ring 26-32, 35392 Giessen, Germany; 3 LOEWE Centre for Translational Biodiversity Genomics (LOEWE-TBG), Senckenberganlage 25, 60325 Frankfurt, Germany; 4 Fraunhofer Institute for Molecular Biology and Applied Ecology, Branch of Bioresources, Ohlebergsweg 12, 35392 Giessen, Germany

**Keywords:** insects, nutrition, food, model organism, animal models

## Abstract

Insects are the most diverse organisms on earth, accounting for ~80% of all animals. They are valuable as model organisms, particularly in the context of genetics, development, behavior, neurobiology and evolutionary biology. Compared to other laboratory animals, insects are advantageous because they are inexpensive to house and breed in large numbers, making them suitable for high-throughput testing. They also have a short life cycle, facilitating the analysis of generational effects, and they fulfil the 3R principle (replacement, reduction and refinement). Many insect genomes have now been sequenced, highlighting their genetic and physiological similarities with humans. These factors also make insects favorable as whole-animal high-throughput models in nutritional research. In this review, we discuss the impact of insect models in nutritional science, focusing on studies investigating the role of nutrition in metabolic diseases and aging/longevity. We also consider food toxicology and the use of insects to study the gut microbiome. The benefits of insects as models to study the relationship between nutrition and biological markers of fitness and longevity can be exploited to improve human health.

## 1. Introduction

Mammalian model organisms have contributed to our understanding of many biological processes, diseases and treatments. They are favored as disease models because they share ~95% of human genes. However, mammals are expensive to house and breed in large numbers, and can be too complex for the analysis of some biological processes [1]. Moreover, the extensive use of mammals raises safety and ethical issues [2] which has resulted in the 3R principle (replacement, reduction and refinement) to limit the use of mammals in research. A suitable alternative must be comparable to mammals (including humans) but must overcome the limitations of mammals in terms of space, cost and ethical restrictions. 

**Figure 1:**
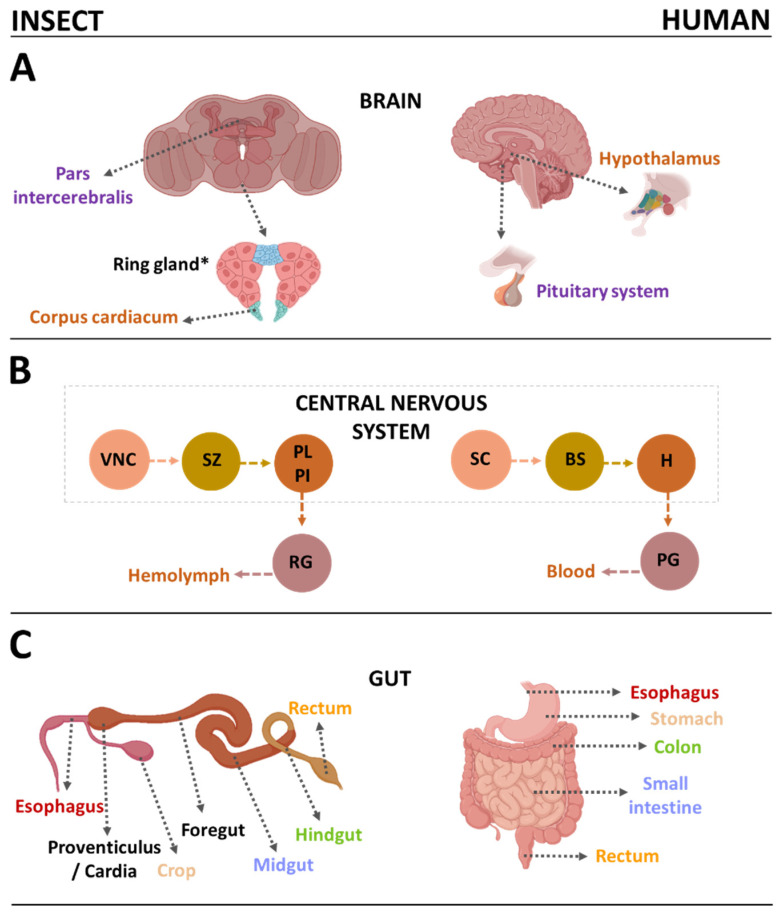
(**A**) Comparison of insect and human brains to show equivalent structures. (**B**) Endocrine pathway in insects and humans (VNC—ventral nerve cord; SZ—subesophageal zone; PI—pars intercerebralis; PL—pars lateralis; RG—ring gland; SC—spinal cord; BS—brainstem; H—hypothalamus; PG—pituitary gland). (**C**) Comparison of insect and human guts to show similar structures. The same colored text represents equivalent organs in insects and humans. * The ring gland is found only in some Dipterans e.g., Drosophila.

**Figure 2:**
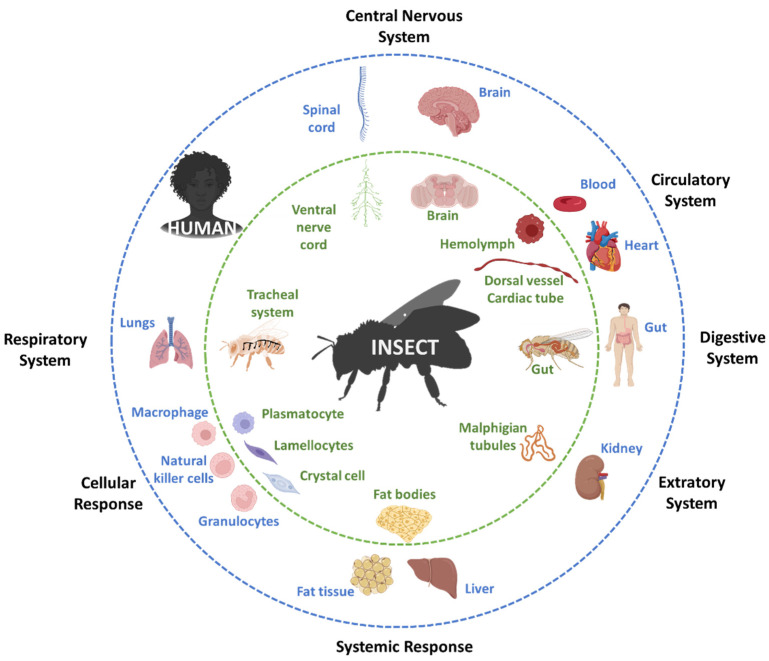
Conserved biological systems in insects and humans.

Insects are well established as model organisms for genetics, development, behavior and neurobiology, but are increasingly considered suitable in medical research because of their genetic and physiological similarities to mammals coupled with only limited ethical restrictions and the ease with which they can be housed and bred in large numbers without sophisticated equipment [3]. Their relatively short life cycle and high reproductive rate also facilitate trans-generational studies and high-throughput screening [4]. The similarities between insects and mammals could allow the widespread use of insects particularly for early-stage preclinical research: for example, ~80% of pathogen infection experiments in mammals could be replaced with insects [5]. The most widely used insect model organisms are the fruit fly *Drosophila melanogaster*, larvae of the greater wax moth *Galleria mellonella* and the silk moth *Bombyx mori*, and the red flour beetle *Tribolium castaneum* [3,6,7,8,9]. Others, used less frequently, include the tobacco hornworm *Manduca sexta*, the seven-spotted ladybeetle *Coccinella septempunctata*, and the common mealworm *Tenebrio molitor* [10,11,12,13,14,15,16]. Comparative genomics has shown that insect genomes contain many homologs of human genes encoding proteins involved in conserved biological mechanisms and pathways. Insects and humans also possess equivalent organs (Figure 1) and similar biological systems (Figure 2). 

The conserved features at the levels of anatomy, physiology, genetics and molecular biology mean that insects are also suitable models for nutritional research. Accordingly, insects have been used to show how macronutrients and micronutrients are transported and metabolized *in vivo*, reflecting the similar physiology of the insect and mammalian gastro-intestinal systems [17]. Insects and mammals also share an evolutionarily conserved innate immune system, providing an opportunity to better understand innate immune responses to food. Insects reared in captivity are often fed on solid diets consisting of complex nutritional sources such as oatmeal or yeast, but holidic diets are increasingly used to allow the precise and reproducible manipulation of food composition [18]. Many factors, including food intake, nutritional composition, locomotor activity, microbiome diversity, fertility, aging and life span can be systematically determined in response to dietary factors [19,20].

In this review, we discuss the value of insects as animal models for the testing of nutrients and dietary components, especially for their effect on lifespan, metabolic and age-related diseases, and aging [21]. Insects can also be used to screen for the health benefits and toxicity of dietary components before testing them in more complex model organisms, and ultimately humans. 

## 2. Model Insects for Nutrition Research

### 2.1. Drosophila Melanogaster (Diptera: Drosophilidae)

The 1.32 × 10^9^ bp genome of the fruit fly *D. melanogaster* [22] is ~20 times smaller than the human genome and contains ~14,000 genes on four chromosome pairs, compared to ~22,000 genes on 23 chromosome pairs in humans. Even so, >60% of human disease genes have homologs or even orthologs in the fruit fly genome [23]. Drosophila mutants can be produced by chemical mutagenesis, irradiation, or the insertion of transposons such as P-elements, targeting more than 80% of its genome [24] and allowing the recovery of mutants within a few weeks [1].

The fruit fly has been a laboratory model for more than a century because it was among the first multicellular organisms used for genetic analysis, most notably in the laboratory of Thomas Hunt Morgan in the early 1900s. Its widespread use has been facilitated, among other properties, by the availability of a large range of genetic markers with easily identified phenotypes, the absence of meiotic recombination in males, and the availability of recessive lethal balancer chromosomes carrying visible markers and multiple inversions to keep stocks of heterozygous lethal alleles. The early use of Drosophila for genetic analysis led to its development as a model for developmental biology, neurobiology, behavioral biology and (most recently) the study of human diseases [25,26]. The latter includes metabolic diseases such as obesity, diabetes and hyperglycemia, as well as diseases associated with aging and longevity, encouraging the use of Drosophila in nutritional research [27]. Although Drosophila has a short lifespan, many of its physiological and molecular aging processes are similar to those in mammals [25]. For example, fruit flies show an age-related decline in motor skills, learning and memory which can be used to study processes related to dementia, Alzheimer’s disease (AD) and Parkinson’s disease (PD) in humans [28]. 

The calorific intake of fruit flies and their consumption of particular nutrients has a significant effect on their lifespan [29,30]. For example, the supplementation of fly food with 200 μM ellagic acid significantly extended the mean and maximum lifespan of male flies compared to those on a control diet, but female flies laid fewer eggs and the time to eclosion fell from 235 to 185 h [31]. Resveratrol, a well-known polyphenolic stilbenoid found in grapes, berries, rhubarb and peanuts [32,33], increased the lifespan and locomotor activity of flies by inducing longevity-associated gene expression [34,35]. Flies fed on a diet containing curcumin were better able to withstand heat stress, increasing their survival [36]. 

In Drosophila, food is digested and absorbed in the crop and midgut, which are equivalent to the human stomach and intestine [26,37]. Indeed, the digestive and neuroendocrine systems of fruit flies are highly conserved in vertebrates and all the key organs in flies that control metabolism have counterparts in humans (Figure 1). For example, the fat bodies are similar to human white fat tissue and liver, Malpighian tubules are similar to human kidneys, oenocytes are similar to human hepatocytes, insulin-producing cells in the pars intercerebralis are similar to islets in the human pancreas, and the corpus cardiacum system is similar to the human hypothalamus-pituitary system [38,39,40]. Accordingly, the Drosophila digestive tract is now at the cutting edge of research [41] and is important for the analysis of obesity-related diseases in humans [42]. There are also biochemical similarities between Drosophila and humans based on a highly conserved set of enzymes that facilitate carbohydrate and lipid metabolism, and hormones that regulate lipogenesis and lipolysis [43]. The energy-sensing and endocrine signaling networks of mammals are also conserved in flies, providing an excellent model for metabolic and diet-associated diseases [44]. 

The use of Drosophila as a model of obesity began in the early 1960s with the isolation of the first obese fly mutant (*adipose*) from a Nigerian wild population. The *adipose* mutants were characterized by excess fat storage and low carbohydrate reserves [45,46]. The *adipose* gene was identified 40 years later [47] and the corresponding protein was found to be structurally and functionally conserved in mammals [48,49]. Drosophila is therefore used as a model to study the effects of high-sugar diets (HSD) and high-fat diets (HFD) on metabolism, gut function, behavior, and aging [50]. Long-term feeding on a HSD and/or HFD leads to obesity in flies, with pathophysiological complications similar to those observed in humans [43,51]. Obesity in flies induced by HFD/HSD is associated with hyperglycemia, cardiomyopathy and a shorter lifespan [27]. Obese flies accumulate triacylglycerols (TAGs), which are the main lipid storage form in flies and humans [43]. The TAGs are stored in the fat body [43] and can be measured in lipids extracted from the fat body or whole fly homogenates [52,53].

**Figure 3:**
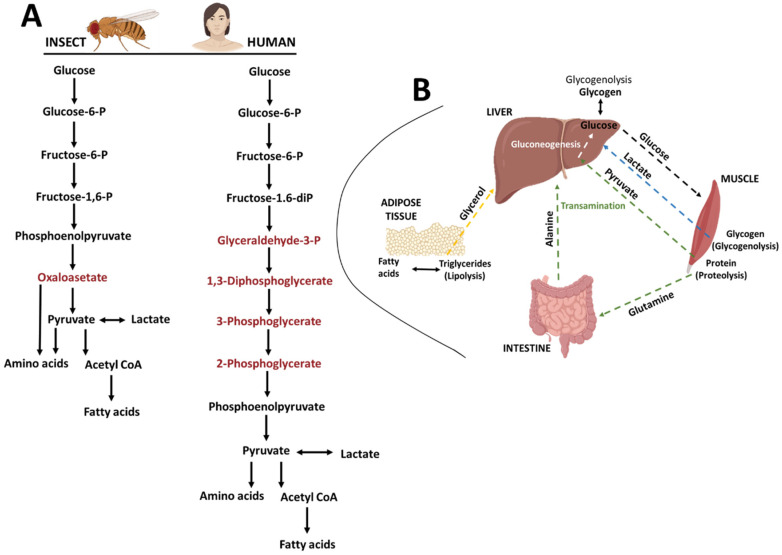
(**A**) Gluconeogenesis pathways in insects and humans (black text indicates the same pathway in both organisms). (**B**) Gluconeogenesis pathway diagram to show interactions between human liver, muscle, intestine and adipose tissues.

Glycolysis is the fundamental pathway for sugar metabolism in all animals [54]. Pyruvate plays a key role in glycolysis and also links to other metabolic pathways in insects and humans (Figure 3). Drosophila fat bodies store glycogen in addition to lipids [39]. The regulation of sugar and fat storage is very similar in flies and humans. The corpus cardiacum secretes adipokinetic hormone (AKH) which is similar to human glucagon [38]. In flies, the pars intercerebralis-corpus cardiacum system manages the physiological activities of numerous peripheral organs [38]. Drosophila can therefore be used as a model to study energy balance, lipid metabolism and glycometabolism [26].

Insulin resistance is a common feature of obesity and type 2 diabetes mellitus (T2DM), which is a complex disease influenced by genetics, the diet and the environment [55]. A Drosophila model of T2DM has been developed with similar pathophysiology to human T2DM, helping to identify gene products and drugs that may improve the outcome for T2DM patients [56]. Drosophila larvae reared on a HSD become hyperglycemic, insulin resistant, and accumulate fat (all indicators of T2DM) when compared with larvae raised on a normal diet [56]. In a recent study, the effects of HSD (30% sucrose) and HFD (15% coconut oil) treatments were linked to the symptoms of obesity and T2DM, including weight gain, the accumulation of glucose and triglycerides, and the abundance of Drosophila insulin-like peptides [51]. The study revealed clear differences in the effects of each diet on survival, glucose and triglyceride levels, and the expression of insulin-like peptides, but both diets induced an obese fly phenotype with linked diseases [51]. Similarly, normal fly food (e.g., yeast and corn starch) with 30% coconut oil (HFD) causes high levels of fat deposition and disrupts insulin/glucose homeostasis [57]. A HFD not only induced obesity but also caused heart dysfunction, which was found to be regulated by the TOR pathway [57,58]. HSD and HFD treatments increase fat storage in flies and also affect carbohydrate-insulin homeostasis, lifespan, locomotor activity, and stress tolerance [59]. 

A HFD also reduces the lifespan and fecundity of flies, and long-term exposure increases AKH transcript levels and enlarges the crop, which stores the excess lipids [50]. Extension of the Drosophila lifespan by dietary restriction (DR) has also been demonstrated [60]. Indeed, DR extends the lifespan of many species, including yeast, nematode, *Daphnia*, and mammals, in addition to insects [61,62]. Even moderate restriction of nutrients drastically alters the mortality of adult Drosophila [62]. Yeast-rich diets inhibit learning performance in old age, whereas low-yeast diets inhibit short-term (5 min) memory in middle age [61]. Conversely, DR enhances the 60-min memory of young flies, and increases their lifespan [61]. 

The composition of the diet for nutritional studies in flies is important because some natural plant-derived compounds affect longevity [29]. For example, rutin (quercetin-3-rutinoside) is a natural flavonol glycoside present in apple, buckwheat, black tea and green tea, and is known for its antioxidant, anti-inflammatory, and anti-diabetic activities [63,64,65,66]. The hormetic efficacy (low-dose effect) of rutin improves longevity and other physiological parameters in Drosophila as part of a standard diet [29]. The presence of 200 or 400 µM rutin significantly improved survival in female and male flies on a HFD, and concentrations > 200 µM significantly improved the climbing efficiency of both sexes and also improved their resistance to heat shock, cold shock, and starvation stress [29]. Black soybean, jaboticaba fruit, many berries, and purple sweet potatoes are rich in antioxidants such as anthocyanin that alter metabolic and inflammatory markers [67,68]. Purple sweet potato anthocyanin (PSPA) reduced the mortality of fruit flies when they were fed a HFD [68]. Real-time PCR revealed that the PSPA supplement upregulated the superoxide dismutase (SOD), catalase (CAT), and Rpn11 (ubiquitin carboxyl-terminal hydrolase) genes compared to the control diet, suggesting that PSPA increases the lifespan of flies by protecting them from oxidative stress [68].

Plant phenols also increase longevity and ameliorate metabolic diseases in insects [69,70,71]. Palm fruit juice (PFJ) from *Elaeis guineensis* contains palm oil phenolics [70,72,73] with beneficial health properties including anti-diabetic activity in mammals by slowing glucose absorption, decreasing insulin resistance and/or improving insulin secretion [70,74]. Different concentrations of PFJ and its fractions were used to assess growth dynamics and anti-aging effects in Drosophila [70]. The study revealed that PFJ extended the growth stages of larvae and increased the lifespan of adult flies [70]. Naringenin is a flavonoid found in grapefruit and tomato that has anti-carcinogenic, anti-inflammatory, anti-estrogenic, anti-hyperlipidemic and anti-hyperglycemic effects in mice, thus inhibiting the formation of fatty deposits in the arteries [75,76,77]. Naringenin also affects longevity, fecundity, resistance to starvation stress, and body weight in male and female fruit flies [75]. The mean lifespan increased in flies fed on a standard diet and a HFD supplemented with up to 400 µM naringenin, whereas concentrations greater than 600 µM were lethal [75]. Female flies on a regular diet supplemented with up to 400 µM naringenin were more resistant to starvation stress than males [75]. Taken together, these results showed that the dose-dependent hormetic efficacy of naringenin varied by sex, diet, and life cycle stage [75].

The testing of several carbohydrates in fruit flies (fructose, glucose and sucrose) revealed that 2–20% sucrose in the diet reduced the lifespan by 13–27%, accompanied by an increase in mortality regardless of age and a significant decline in fecundity [78]. Sucrose is commonly used in Drosophila laboratory food but may shorten the lifespan and, when combined with a low dietary protein content, also reduce egg-laying capability [78]. A lower protein to carbohydrate (P:C) ratio increased the life span of the flies. Increasing the P:C ratio shortened the lifespan by inducing an age-dependent increase in mortality, whereas the effect was weaker when the P:C ratio was reduced [79]. 

A balanced diet must contain sufficient quantities of vitamins (essential organic micronutrients) and minerals (essential inorganic nutrients) to maintain health [80]. A lack of micronutrients caused by an unbalanced diet, malabsorption or other factors such as pregnancy may lead to deficiency-related diseases [81,82,83] but excess vitamin consumption can also be harmful [80]. Several studies have addressed the effects of vitamins using insect models [84,85,86,87,88]. For example, insufficient amounts of the water-soluble vitamin B7 (biotin) are associated with fetal malformation and neurological disorders in humans [89,90,91,92]. Propionyl-CoA carboxylase (PCC) catalyzes the carboxylation of propionyl-CoA to methylmalonyl-CoA, and loss of function leads to inherited metabolic disorders in humans due to biotin deficiency [93]. The effect of biotin on lifespan, stress tolerance and fertility was studied in Drosophila, revealing lower PCC activity and biotin levels in males than females when they were fed on biotin-deficient diets [90]. The lifespan of both sexes was reduced by ~30% due to biotin deficiency, which also reduced fertility as shown by the lower egg hatching rate [90]. 

Nutrigenomics (nutritional genomics) is the study of gene-nutrient interactions and how they affect the health and metabolism of an organism, which may facilitate the development of personalized nutritional interventions [94,95]. The combination of nutrigenomics with longevity studies helps to determine the impact of nutrients on aging and life span [96]. Drosophila larvae and adults were fed on control diets or diets containing high levels of saturated fat palmitic acid, soy or 95% lean ground beef [96]. Remarkably, triglyceride and total protein levels declined in adult flies on the beef diet, and total protein levels were higher in males fed on the soy diet, but all diets significantly decreased the longevity of male and female flies [96]. Microarray analysis of adult flies on the different diets showed that only 2–3% of the ~18,000 genes were influenced by the diet [96]. Drosophila is therefore a valuable model in which to combine nutrigenomics with the analysis of longevity and metabolic diseases in relation to nutrition [59,96]. 

### 2.2. Tribolium Castaneum (Coleoptera: Tenebrionidae)

The red flour beetle is a cosmopolitan insect pest of stored grains, which has evolved to utilize food sources varying in nutritional quality [97]. Tribolium is therefore used to study nutritional genomics, particularly with regard to longevity and stress tolerance [98]. The beetles can be reared on flour in small containers at 30 °C without an external water supply [8,99]. The developmental cycle is completed within 4 weeks, and adults can live up to 3 years [99]. The *T. castaneum* genome has been sequenced [100], revealing a greater degree of conservation between Tribolium and human genes than is the case for other model insects [98]. 

Tribolium beetles are ideal to study the impact of complex diets on longevity, allowing the identification of food–gene interactions that affect stress tolerance [101]. For example, 1% lyophilized broccoli added to flour increased the lifespan of beetles reared at 32 °C and also protected them from heat stress (42 °C) compared to beetles on a control diet of normal flour [101]. The genes involved in stress tolerance included homologs of *Nrf-2*, *Jnk-1* and *Foxo-1*, which were shown by RNA interference (RNAi) to be responsible for the effects on longevity [101]. Tribolium has also been used to investigate the effect of complex nutrition on lifespan by assessing the survival of the beetles under heat stress (42 °C) when fed on different diets. The addition of environmental carcinogens and the food contaminant benz(a)pyrene to flour reduced the lifespan of the beetles [102], whereas the addition of a red wine extract extended the lifespan [98].

Tribolium beetles have also been used as a model to explore the impact of nutritional quality on male development and reproduction. Males developed faster and their body size was larger on a higher-quality diet, but mating, insemination rate, and reproductive success in a population context were unaffected [97]. The diet quality therefore affected male larval development but not adult reproductive performance [97]. The addition of nutrient-free fillers to the diet affected synthesis of the pheromone 4,8-dimethyldecanal (DMD), a condition-dependent mating signal that increases the olfactory attractiveness of adult males [103]. Males on a high-quality diet produced three times more DMD than those on a low-quality diet. The authors proposed that DMD production rates depend on the nutritional quality of food, although it was not clear whether there was any overall effect on the ability of males to attract females [103].

Probiotics are live microorganisms that, when consumed in appropriate amounts, promote health by modulating the immune system [104,105]. The most common probiotics are the bacterial genera *Lactobacillus*, *Bifidobacterium* and *Streptococcus*, as well as the yeast genus *Saccharomyces* [105]. Tribolium beetles have been used as a model organism to characterize *in vivo* the probiotic *Enterococcus mundtii* isolated from the larval feces of the Mediterranean flour moth *Ephestia kuehniella* [106]. Tribolium larvae were fed with the probiotic isolate or the corresponding supernatant before infection with an entomopathogen: *Bacillus thuringiensis* or *Pseudomonas entomophila* [106]. Larvae exposed to the isolate/supernatant were more likely to survive following infection with *B. thuringiensis*, but not with *P. entomophila* [106]. These results suggest that Tribolium beetles could be suitable for the pre-screening of probiotics [106]. Tribolium beetles are already used for food safety and functionality studies, which are needed to reduce both economic losses and the threat to consumer health [107].

### 2.3. Galleria Mellonella (Lepidoptera: Pyralidae)

The development of the greater wax moth is regulated by the ambient temperature and takes only 8–12 weeks under optimum conditions [108,109,110]. Females can lay ~1500 eggs [3,109]. The larvae have several features in common with mammals and accordingly they have been used in many studies as alternatives to mammalian models [3,111,112,113,114,115,116]. The *G. mellonella* genome has also been sequenced, making it easier to find genes associated with nutrition [117]. 

Given the association between food quantity/quality and health [118], Galleria larvae have been used to study the impact of (i) a poor-quality diet and (ii) an artificial parasite-like immune challenge on pupal development, growth and adult immunity. A hostile nutritional environment can increase the risk of infection, thus provoking more frequent and robust immune responses, ultimately leading to greater fitness [119]. Categories of food differing in nutritional quality therefore affect the life history traits of Galleria [120]. For example, larvae fed on a low-quality diet were more susceptible to infection by the yeast *Candida albicans*, and the abundance of several antimicrobial peptides (AMPs) in the hemolymph declined [121]. This clearly showed that a poor diet inhibits the immune response and increases susceptibility to infection. Importantly, larvae provided with high-energy food grew rapidly, but had weak immunity and the highest mortality rates, whereas those provided with average nutrition took longer to develop but had a stronger immune system. The group provided with low-energy food also took longer to develop but they had strong immunity and the lowest mortality rates [120]. Maternal nutrition was also shown to directly influence the immunity of Galleria larvae [122]. 

Probiotic microbes stimulate the immune system and protect against pathogens [123,124,125]. Several studies highlight the use of Galleria larvae to screen the probiotic activity of bacteria and also the importance of probiotics in the response to bacterial infections [123,124,126,127,128,129,130].

### 2.4. Other Insects

Although Drosophila, Tribolium and Galleria are the most widely used insect models for nutritional studies, other species have been used when they are suitable for the investigation of specific compounds. For example, the effect of lifetime astaxanthin supplementation on longevity was studied using the mealworm beetle *Tenebrio molitor* (Coleoptera: Tenebrionidae) [131]. Astaxanthin is a red-orange carotenoid pigment that is mostly found in fresh water microalgae and seafood [132,133,134]. It is a powerful antioxidant that has been used to treat metabolic, neurodegenerative, inflammatory, and age-related diseases [131,135]. Astaxanthin reduced the growth rate, immunity and survival of larvae that were not challenged with a pathogen, but limited the immunopathological cost of survival [131].

The effect of different diets on longevity has also been tested in the Mediterranean medfly *Ceratitis capitate* (Diptera: Tephritidae) [136]. DR affected the cost of reproduction in both sexes. Female medflies lived longer than male medflies regardless of the diet, revealing no significant interaction between diet and sex in the determination of lifespan [136]. The effects of DR on the lifespan of the oriental fruit fly *Bactrocera dorsalis* (Diptera: Tephritidae) were investigated by feeding them on different ratios of yeast and sugar at 6-day intervals [137]. The lifespan of the flies was extended by DR, and lifespan was also affected by the carbohydrate content of the diet [137]. Female flies lived longer than males regardless of the diet, again suggesting there is no significant interaction between diet and sex in the determination of lifespan, at least for the diets tested in this study [137]. 

Caloric restriction (CR) is defined as the restriction of energy intake without malnutrition, and is widely associated with health and longevity [138,139]. The influence of calories and nutrients on lifespan and fecundity was tested in unmated females of the Queensland fruit fly *Bactocera tryoni* (Diptera: Tephritidae) using diets varying in P:C ratios and concentrations. The study revealed that nutrients rather than CR have the main impact on lifespan [140].

Early studies investigated the importance of nutrition on the reproductive system, for example in male bush crickets (Orthoptera: Tettigoniidae: *Requena verticalis* Walker). Male crickets fed on a low-quality diet showed fewer mating trials than those provided with a high-quality diet [141]. Similarly, the male reproductive system of the Hawaiian fly *Drosophila grimshawi* Oldenberg (Diptera: Drosophilidae) was directly affected by the diet [142]. When males of the black blow fly (Diptera: Calliphoridae: *Phormia regina* Meigen) were fed on a low-protein diet, they inseminated fewer females [143]. Finally, a study of the scorpionfly *Panorpa nuptialis* Gerstaecker (Mecoptera: Panorpidae) showed that well-fed males copulated more frequently than those provided with a poor diet [144].

## 3. Comparison of the Vertebrate and Invertebrate Gut Microbiome

Gut bacteria have a direct impact on the development, fecundity, immunity, and lifespan of their host, and a healthy microbiome is therefore beneficial [145,146,147,148,149,150]. The human gut microbiome varies from person to person, but the core bacterial taxa are relatively stable. Even so, the composition, abundance and function of different bacteria change during aging [146,151], and the age-related disruption of the gut microbiome can affect health and lead to a shorter lifespan [152]. The underlying mechanisms are unclear, and better models are therefore required [153]. Drosophila is a valuable insect model to study the interaction between gut microbes, the immune system and aging in humans because the Drosophila gastrointestinal tract is similar to its mammalian counterpart [146,151]. Impressively, the characterization of microbes and regenerative stem cells in the Drosophila gut has led to the use of Drosophila as a model to study the regulation of the gut epithelium and its microbiome [154]. Although Drosophila gut microbes do not mimic the human gut microbiome directly, they are sufficient to investigate how the microbiome affects health and behavior [155,156], facilitated by the genetic tools and resources available in Drosophila [153]. For example, Drosophila hosts only a small number of bacterial populations in its gut, including species also present in the human microbiome [157]. This provides a simplified model to study host–microbiome interactions [158]. In insects, the gut microbiome plays a key role in host nutritional physiology [159]. The gut microbiome regulates gut homeostasis in Drosophila to maintain fitness and a normal lifespan, whereas changes in microbial composition promote aging [158]. Insects can therefore be used as tools to determine how to manipulate the gut microbiome and maintain health [160,161]. The gut microbiome contributes to age-associated cellular and physiological changes in the intestine and, also to age-related changes in the gut microbiome itself which may affect the health of older fruit flies [160]. Interestingly, the removal of commensal microbes increased the lifespan of Drosophila, suggesting that there is a cost associated with the normal bacterial load [149].

Neurodegenerative disorders including AD and PD are the main causes of dementia in the elderly human population [162,163,164,165]. The gut microbiome is involved not only in nutrient metabolism but also in the onset and progression of neurodegenerative diseases [155,166]. Dysbiosis in the Drosophila gut reduces the lifespan, inhibits motor function, and causes neurodegeneration in AD and PD models [155]. The potential of *Lactobacillus* probiotics to reverse the progression of AD has been demonstrated in a Drosophila AD model [166]. The gut microbiome in the AD model is characterized by a greater abundance of *Wolbachia* than normal flies [166]. However, the administration of *Lactobacillus plantarum* DR7 improved the diversity of the microbiome and reduced the abundance of *Wolbachia* [166]. This may be a promising therapeutic approach because the abundance of *Wolbachia* is positively correlated with neurodegenerative disorders in the fruit fly, including PD, AD and Huntington’s disease [166]. The analysis of transgenic GMR-Aβ42 flies, which mimic AD, showed the potential of *Lactobacillus* spp. to prevent or delay the onset and development of neurodegenerative diseases [167]. 

The dysregulation of gut–microbe interactions also causes gut inflammation [168] that can progress to chronic inflammatory diseases or even gastrointestinal cancer [169]. However, the molecular mechanisms that underlie gut–microbe homeostasis and pathogenesis are poorly understood [168,169]. Drosophila has also been used as a model of inflammatory diseases [169]. The interactions between gut bacteria and host immunity have been investigated in the context of bacteria that can activate the intestinal dual oxidase system in Drosophila, which may lead to a better understanding of the biology of inflammatory diseases in humans [168]. 

Other insects have also been used to study diet–microbiome interactions. For example, the Tribolium gut microbiome is derived from flour-acquired microbes, and varies depending on the nature of the flour resource and the population density [170]. The microbiome confers fitness benefits such as higher fecundity/egg survival and a greater lifespan, while reducing the frequency of cannibalism. Importantly, the microbiome was not required for beetle development or survival under any of the tested conditions [170]. The induction of obesity and other metabolic disorders by a HFD is also associated with an alteration of the gut microbiome, as recently demonstrated in the honey bee *Apis mellifera* [171]. Excess dietary fat (mainly palm oil) and the resulting weight gain shortened the lifespan, induced hyperglycemia and caused the accumulation of fat in the bees, but the absence of a microbiome did not have a significant effect; the HFD also increased the relative abundance of Gilliamella at the expense of Bartonella [171]. In another study, 16S rRNA amplicon sequencing in different Galleria tissues showed very little diversity, supporting the potential of the larvae as a model host system to study host–microbiome interactions in detail [172].

## 4. Insects in Food Toxicological Studies

The toxicological analysis of food additives and ingredients is necessary to avoid human exposure to potentially harmful compounds in food [173,174,175]. Although mammals are traditionally used for this purpose, several studies have evaluated the toxicity of food-borne toxins in insect models. For example, acrylamide can form when food is heated [176], causing the amino group of asparagine to react with the carbonyl group of a sugar via the Maillard reaction [177]. Acrylamide is hepatoxic, carcinogenic and neurotoxic [178,179,180,181,182,183]. EU regulations mandate the monitoring of foods for acrylamide, contributing to significant reductions throughout the food chain [184,185]. Tribolium beetles have been used as a model to assess the effects of acrylamide on fitness and survival, and their influence on transcription factors Nrf-2 and Ahr, which regulate genes involved in phase I and II xenobiotic metabolism [186]. Acrylamide (0.5–10%) was added to the standard flour diet and the beetles were maintained on the diet at 32 °C for more than 2 weeks, resulting in a significant dose-dependent decrease in fitness and survival, which was exacerbated when RNAi was used to knock down the *Ahr* and *Nrf-2* genes [186]. 

In a similar study, the beetles were tested for survival under heat stress after feeding on charred toast [180]. Beetles fed solely on charred toast died much earlier than beetles fed on control flour, whereas beetles fed on flour enriched with 5% charred toast survived significantly longer than the controls [180]. Knocking down the *Ahr* and *Nrf-2* genes increased the susceptibility of beetles when they were fed solely on charred toast [180]. 

Numerous studies show the effects of food-borne toxins on insects. Okadaic acid is a potent polyether marine toxin produced mainly by mollusks and fish, which causes diarrheic shellfish poisoning in humans [187,188,189]. The cellular and biochemical effects of okadaic acid on Galleria were tested by feeding or injecting larvae with okadaic acid [190]. The injection of ≥75 ng okadaic acid per larva reduced larval survival by more than 65%, and was associated with a >50% decrease in the number of circulating hemocytes [190]. Force-feeding Galleria with okadaic acid also increased oxidative damage in the midgut. Eight common food preservatives (potassium nitrate, potassium nitrite, potassium sorbate, sodium benzoate, sodium nitrate, sodium chloride, sodium nitrite, and sodium acetate) were also administered to Galleria larvae by injection and force-feeding to measure the toxicity of these compounds [191]. 

The filamentous fungus *Aspergillus fumigatus* is the most important life-threatening opportunistic fungal pathogen of humans and animals [192,193]. The presence of this fungus or its toxin (fumagillin) in food can be fatal for immunocompromised individuals [192,194]. There are several studies showing *A. fumigatus* toxicity in insect models [195,196,197]. Galleria larvae were used to evaluate the toxicity of fumagillin, which increased their susceptibility to subsequent infections with *A. fumigatus* conidia by reducing the ability of hemocytes to kill opsonized *Candida albicans* cells and to phagocytose *A. fumigatus* conidia. This work demonstrated that fumagillin suppresses the immune response of Galleria larvae [198].

The regular consumption of ethanol in alcoholic beverages and as a solvent in herbal medicines has a negative effect on humans, causing liver disorders, cardiovascular disorders, neuropsychic disorders and reprotoxicity [199,200]. Ethanol behaves like a toxin, affecting behavior and the host microbiome [201]. The effect of ethanol was studied in a Drosophila model of chronic, non-intoxicating ethanol ingestion, and was shown to affect feeding behavior and triglyceride levels among other factors [201]. 

Drosophila has also been used to evaluate the toxicity of nanoparticles [202], which are widely used in medicine and food preservation but can have harmful effects [203,204]. In this context, Drosophila is favored as an *in vivo* toxicity model for high-throughput testing [203,205,206,207,208,209,210]. In one study, several types of nanoparticles were shown to delay development, reduce the number of pupae and hatched eggs, and to cause weight loss [204]. Adult flies treated with nanoparticles as larvae showed sensory and locomotor defects that altered their behavioral phenotypes [204]. Another study assessed the toxicity of orally-delivered titanium dioxide nanoparticles on the survival, life cycle and mechanosensory behavior of Drosophila, and the structure of various mechanosensory organs [211]. These nanoparticles promoted the generation of reactive oxygen species, which influenced signaling pathways controlling the development and behavior of the flies [211]. Drosophila was also used to evaluate the toxicity of food-grade titanium dioxide (E171) by exposing larvae to different concentrations of the nanoparticles and then assessing parameters such as survival, fecundity, and pupation time. Titanium dioxide did not affect survival or fecundity, but significantly increased the time to pupation. Ultimately, titanium dioxide showed limited toxicity to Drosophila at concentrations relevant to human oral exposure [212].

## 5. Conclusions

The use of mammals as research models is expensive and subject to strict ethical regulations that reduce the number of individuals used in any experiment to the absolute minimum required to achieve sufficient statistical power during data analysis. Insects overcome these drawbacks because they are inexpensive to rear and maintain under laboratory conditions, and they are not subject to ethical limitations according to the animal protection law. The genetic and physiological properties of insects are also similar enough to mammals to allow the replacement of mammals in many experiments, particularly those in the early stages of preclinical development. Insects are particularly suitable for the investigation of molecular mechanisms that contribute to longevity and its correlation with the aging process and nutrition. They can also be used to screen dietary additives and contaminants to establish their health benefits or toxicities. Although insects provide a high-throughput in vivo model, it is still essential to combine them with mammalian models and human studies. Overall, these small organisms provide robust model systems that serve as a foundation to reveal conserved aspects related to human nutrition, including the link with metabolic, age-related and neurodegenerative diseases, and the function of the gut microbiome. 

## Data Availability

Not applicable.

## Abbreviations

AD	Alzheimer’s disease
AKH	adipokinetic hormone
AMP	antimicrobial peptide
BS	brain stem
CAT	catalase
CR	caloric restriction
DMD	pheromone 4,8-dimethyl decanal
DR	dietary restriction
H	hypothalamus
HFD	high fat diet
HSD	high sugar diet
P:C	protein:carbohydrate
PCC	propionyl-CoA carboxylase
PD	Parkinson’s disease
PFJ	palm fruit juice
PG	pituitary gland
PI	pars intercerebralis
PL	pars lateralis
PSPA	purple sweet potato anthocyanin
RG	ring gland
RNAi	RNA interference
Rpn11	ubiquitin carboxyl-terminal hydrolase
SC	spinal cord
SOD	superoxide dismutase
SZ	subesophageal zone
TAG	triacylglycerol
T2DM	type 2 diabetes mellitus
VNC	ventral nerve cord

## References

[1] Sandner G., König A., Wallner M., Weghuber J. (2022). Alternative Model Organisms for Toxicological Fingerprinting of Relevant Parameters in Food and Nutrition. Crit. Rev. Food Sci. Nutr..

[2] Levy N. (2012). The Use of Animal as Models: Ethical Considerations. Int. J. Stroke.

[3] Mikulak E., Gliniewicz A., Przygodzka M., Solecka J. (2018). *Galleria mellonella* L. as Model Organism Used in Biomedical and Other Studies. Prz. Epidemiol..

[4] Jans K., Lüersen K., Rimbach G. (2021). *Drosophila melanogaster* as a Model Organism to Study Lithium and Boron Bioactivity. Int. J. Mol. Sci..

[5] Renwick J., Kavanagh K. (2007). Insects as models for studying the virulence of fungal pathogens of humans. New Insights in Medical Mycology.

[6] Abdelli N., Peng L., Keping C. (2018). Silkworm, Bombyx Mori, as an Alternative Model Organism in Toxicological Research. Environ. Sci. Pollut. Res. Int..

[7] Meng X., Zhu F., Chen K. (2017). Silkworm: A Promising Model Organism in Life Science. J. Insect Sci..

[8] Rösner J., Wellmeyer B., Merzendorfer H. (2020). Tribolium Castaneum: A Model for Investigating the Mode of Action of Insecticides and Mechanisms of Resistance. Curr. Pharm. Des..

[9] Yamaguchi M., Yoshida H. (2018). Drosophila as a Model Organism. Adv. Exp. Med. Biol..

[10] de Carvalho N.M., Teixeira F., Silva S., Madureira A.R., Pintado M.E. (2019). Potential Prebiotic Activity of Tenebrio Molitor Insect Flour Using an Optimized in Vitro Gut Microbiota Model. Food Funct..

[11] Gershman A., Romer T.G., Fan Y., Razaghi R., Smith W.A., Timp W. (2021). De Novo Genome Assembly of the Tobacco Hornworm Moth (*Manduca sexta*). G3 Genes|Genomes|Genet.

[12] Jo Y.H., Lee J.H., Patnaik B.B., Keshavarz M., Lee Y.S., Han Y.S. (2021). Autophagy in Tenebrio Molitor Immunity: Conserved Antimicrobial Functions in Insect Defenses. Front. Immunol..

[13] Lozoya-Pérez N.E., García-Carnero L.C., Martínez-Álvarez J.A., Martínez-Duncker I., Mora-Montes H.M. (2021). Tenebrio Molitor as an Alternative Model to Analyze the Sporothrix Species Virulence. Infect. Drug Resist..

[14] Lyons N., Softley I., Balfour A., Williamson C., O’Brien H.E., Shetty A.C., Bruno V.M., Diezmann S. (2020). Tobacco Hornworm (*Manduca sexta*) Caterpillars as a Novel Host Model for the Study of Fungal Virulence and Drug Efficacy. Virulence.

[15] Miao Z., Cao X., Jiang H. (2020). Digestion-Related Proteins in the Tobacco Hornworm, *Manduca sexta*. Insect Biochem. Mol. Biol..

[16] Ren X.Y., Zhang L.S., Han Y.H., An T., Liu Y., Li Y.Y., Chen H.Y. (2016). Proteomic Research on Diapause-Related Proteins in the Female Ladybird, *Coccinella septempunctata* L. Bull. Entomol. Res..

[17] Rubio-Aliaga I. (2012). Model Organisms in Molecular Nutrition Research. Mol. Nutr. Food Res..

[18] Piper M.D.W., Blanc E., Leitão-Gonçalves R., Yang M., He X., Linford N.J., Hoddinott M.P., Hopfen C., Soultoukis G.A., Niemeyer C. (2014). A Holidic Medium for *Drosophila melanogaster*. Nat. Methods.

[19] Gimeno-Mallench L., Sanchez-Morate E., Parejo-Pedrajas S., Mas-Bargues C., Inglés M., Sanz-Ros J., Román-Domínguez A., Olaso G., Stromsnes K., Gambini J. (2020). The Relationship between Diet and Frailty in Aging. Endocr. Metab. Immune Disord. Drug Targets.

[20] Staats S., Lüersen K., Wagner A.E., Rimbach G. (2018). *Drosophila melanogaster* as a Versatile Model Organism in Food and Nutrition Research. J. Agric. Food Chem..

[21] Brandt A., Vilcinskas A. (2013). The Fruit Fly *Drosophila melanogaster* as a Model for Aging Research. Adv. Biochem. Eng. Biotechnol..

[22] Adams M.D., Celniker S.E., Holt R.A., Evans C.A., Gocayne J.D., Amanatides P.G., Scherer S.E., Li P.W., Hoskins R.A., Galle R.F. (2000). The Genome Sequence of *Drosophila melanogaster*. Science.

[23] Rubin G.M., Yandell M.D., Wortman J.R., Gabor Miklos G.L., Nelson C.R., Hariharan I.K., Fortini M.E., Li P.W., Apweiler R., Fleischmann W. (2000). Comparative Genomics of the Eukaryotes. Science.

[24] Parks A.L., Cook K.R., Belvin M., Dompe N.A., Fawcett R., Huppert K., Tan L.R., Winter C.G., Bogart K.P., Deal J.E. (2004). Systematic Generation of High-Resolution Deletion Coverage of the *Drosophila melanogaster* Genome. Nat. Genet..

[25] De Nobrega A.K., Lyons L.C. (2020). Aging and the Clock: Perspective from Flies to Humans. Eur. J. Neurosci..

[26] Smith W.W., Thomas J., Liu J., Li T., Moran T.H. (2014). From Fat Fruitfly to Human Obesity. Physiol. Behav..

[27] Gáliková M., Klepsatel P. (2018). Obesity and Aging in the Drosophila Model. Int. J. Mol. Sci..

[28] Poetini M.R., Araujo S.M., Trindade de Paula M., Bortolotto V.C., Meichtry L.B., Polet de Almeida F., Jesse C.R., Kunz S.N., Prigol M. (2018). Hesperidin Attenuates Iron-Induced Oxidative Damage and Dopamine Depletion in *Drosophila melanogaster* Model of Parkinson’s Disease. Chem.-Biol. Interact..

[29] Chattopadhyay D., Thirumurugan K. (2020). Longevity-Promoting Efficacies of Rutin in High Fat Diet Fed *Drosophila melanogaster*. Biogerontology.

[30] Savola E., Montgomery C., Waldron F.M., Monteith K.M., Vale P., Walling C. (2021). Testing Evolutionary Explanations for the Lifespan Benefit of Dietary Restriction in Fruit Flies (*Drosophila melanogaster*). Evolution.

[31] Kharat P., Sarkar P., Mouliganesh S., Tiwary V., Priya V.B.R., Sree N.Y., Annapoorna H.V., Saikia D.K., Mahanta K., Thirumurugan K. (2020). Ellagic Acid Prolongs the Lifespan of *Drosophila melanogaster*. GeroScience.

[32] Galiniak S., Aebisher D., Bartusik-Aebisher D. (2019). Health Benefits of Resveratrol Administration. Acta Biochim. Pol..

[33] Malaguarnera L. (2019). Influence of Resveratrol on the Immune Response. Nutrients.

[34] Chandrashekara K.T., Shakarad M.N. (2011). Aloe Vera or Resveratrol Supplementation in Larval Diet Delays Adult Aging in the Fruit Fly, *Drosophila melanogaster*. J. Gerontol. A Biol. Sci. Med. Sci..

[35] Staats S., Wagner A.E., Kowalewski B., Rieck F.T., Soukup S.T., Kulling S.E., Rimbach G. (2018). Dietary Resveratrol Does Not Affect Life Span, Body Composition, Stress Response, and Longevity-Related Gene Expression in *Drosophila melanogaster*. Int. J. Mol. Sci..

[36] Chen Y., Liu X., Jiang C., Liu L., Ordovas J.M., Lai C.-Q., Shen L. (2018). Curcumin Supplementation Increases Survival and Lifespan in Drosophila under Heat Stress Conditions. BioFactors.

[37] Pitsouli C., Perrimon N. (2008). Developmental Biology: Our Fly Cousins’ Gut. Nature.

[38] Bader R., Colomb J., Pankratz B., Schröck A., Stocker R.F., Pankratz M.J. (2007). Genetic Dissection of Neural Circuit Anatomy Underlying Feeding Behavior in Drosophila: Distinct Classes of Hugin-Expressing Neurons. J. Comp. Neurol..

[39] Canavoso L.E., Jouni Z.E., Karnas K.J., Pennington J.E., Wells M.A. (2001). Fat Metabolism in Insects. Annu. Rev. Nutr..

[40] Gutierrez E., Wiggins D., Fielding B., Gould A.P. (2007). Specialized Hepatocyte-like Cells Regulate Drosophila Lipid Metabolism. Nature.

[41] Lemaitre B., Miguel-Aliaga I. (2013). The Digestive Tract of *Drosophila melanogaster*. Annu. Rev. Genet..

[42] Miguel-Aliaga I., Jasper H., Lemaitre B. (2018). Anatomy and Physiology of the Digestive Tract of *Drosophila melanogaster*. Genetics.

[43] Musselman L.P., Kühnlein R.P. (2018). Drosophila as a Model to Study Obesity and Metabolic Disease. J. Exp. Biol..

[44] Owusu-Ansah E., Perrimon N. (2014). Modeling Metabolic Homeostasis and Nutrient Sensing in Drosophila: Implications for Aging and Metabolic Diseases. Dis. Models Mech..

[45] Doane W.W. (1961). Developmental Physiology of the Mutant Female Sterile(2)Adipose of *Drosophila melanogaster*. III. Corpus Allatum-Complex and Ovarian Transplantations. J. Exp. Zool..

[46] Doane W.W. (1960). Developmental Physiology of the Mutant Female Sterile(2)Adipose of *Drosophila melanogaster*. I. Adult Morphology, Longevity, Egg Production, and Egg Lethality. J. Exp. Zool..

[47] Häder T., Müller S., Aguilera M., Eulenberg K.G., Steuernagel A., Ciossek T., Kühnlein R.P., Lemaire L., Fritsch R., Dohrmann C. (2003). Control of Triglyceride Storage by a WD40/TPR-Domain Protein. EMBO Rep..

[48] Lai C.-Q., Parnell L.D., Arnett D.K., García-Bailo B., Tsai M.Y., Kabagambe E.K., Straka R.J., Province M.A., An P., Borecki I.B. (2009). WDTC1, the Ortholog of Drosophila Adipose Gene, Associates With Human Obesity, Modulated by MUFA Intake. Obesity.

[49] Suh J.M., Zeve D., McKay R., Seo J., Salo Z., Li R., Wang M., Graff J.M. (2007). Adipose Is a Conserved Dosage-Sensitive Antiobesity Gene. Cell Metab..

[50] Liao S., Amcoff M., Nässel D.R. (2021). Impact of High-Fat Diet on Lifespan, Metabolism, Fecundity and Behavioral Senescence in Drosophila. Insect Biochem. Mol. Biol..

[51] Baenas N., Wagner A.E. (2022). *Drosophila melanogaster* as a Model Organism for Obesity and Type-2 Diabetes Mellitus by Applying High-Sugar and High-Fat Diets. Biomolecules.

[52] Al-Anzi B., Sapin V., Waters C., Zinn K., Wyman R.J., Benzer S. (2009). Obesity-Blocking Neurons in Drosophila. Neuron.

[53] Carvalho M., Sampaio J.L., Palm W., Brankatschk M., Eaton S., Shevchenko A. (2012). Effects of Diet and Development on the Drosophila Lipidome. Mol. Syst. Biol..

[54] Chaudhry R., Varacallo M. (2022). Biochemistry, Glycolysis. StatPearls.

[55] Mastrototaro L., Roden M. (2021). Insulin Resistance and Insulin Sensitizing Agents. Metabolism.

[56] Palanker Musselman L., Fink J.L., Narzinski K., Ramachandran P.V., Sukumar Hathiramani S., Cagan R.L., Baranski T.J. (2011). A High-Sugar Diet Produces Obesity and Insulin Resistance in Wild-Type Drosophila. Dis. Models Mech..

[57] Birse R.T., Choi J., Reardon K., Rodriguez J., Graham S., Diop S., Ocorr K., Bodmer R., Oldham S. (2010). High-Fat-Diet-Induced Obesity and Heart Dysfunction Are Regulated by the TOR Pathway in Drosophila. Cell Metab..

[58] Oldham S. (2011). Obesity and Nutrient Sensing TOR Pathway in Flies and Vertebrates: Functional Conservation of Genetic Mechanisms. Trends Endocrinol. Metab..

[59] Eickelberg V., Lüersen K., Staats S., Rimbach G. (2022). Phenotyping of *Drosophila melanogaster*-A Nutritional Perspective. Biomolecules.

[60] Tatar M. (2007). Diet Restriction in *Drosophila melanogaster*. Design and Analysis. Interdiscip. Top Gerontol..

[61] Burger J.M.S., Buechel S.D., Kawecki T.J. (2010). Dietary Restriction Affects Lifespan but Not Cognitive Aging in *Drosophila melanogaster*. Aging Cell.

[62] Pletcher S.D., Libert S., Skorupa D. (2005). Flies and Their Golden Apples: The Effect of Dietary Restriction on Drosophila Aging and Age-Dependent Gene Expression. Ageing Res. Rev..

[63] Budzynska B., Faggio C., Kruk-Slomka M., Samec D., Nabavi S.F., Sureda A., Devi K.P., Nabavi S.M. (2019). Rutin as Neuroprotective Agent: From Bench to Bedside. Curr. Med. Chem..

[64] Chua L.S. (2013). A Review on Plant-Based Rutin Extraction Methods and Its Pharmacological Activities. J. Ethnopharmacol..

[65] Motallebi M., Khorsandi K., Sepahy A.A., Chamani E., Hosseinzadeh R. (2020). Effect of Rutin as Flavonoid Compound on Photodynamic Inactivation against P. Aeruginosa and S. Aureus. Photodiagn. Photodyn. Ther..

[66] Negahdari R., Bohlouli S., Sharifi S., Maleki Dizaj S., Rahbar Saadat Y., Khezri K., Jafari S., Ahmadian E., Gorbani Jahandizi N., Raeesi S. (2021). Therapeutic Benefits of Rutin and Its Nanoformulations. Phytother. Res..

[67] Lee Y.-M., Yoon Y., Yoon H., Park H.-M., Song S., Yeum K.-J. (2017). Dietary Anthocyanins against Obesity and Inflammation. Nutrients.

[68] Wang L., Li Y.M., Lei L., Liu Y., Wang X., Ma K.Y., Zhang C., Zhu H., Zhao Y., Chen Z.-Y. (2016). Purple Sweet Potato Anthocyanin Attenuates Fat-Induced Mortality in *Drosophila melanogaster*. Exp. Gerontol..

[69] Bass T.M., Weinkove D., Houthoofd K., Gems D., Partridge L. (2007). Effects of Resveratrol on Lifespan in *Drosophila melanogaster* and Caenorhabditis Elegans. Mech. Ageing Dev..

[70] Leow S.-S., Luu A., Shrestha S., Hayes K.C., Sambanthamurthi R. (2018). Drosophila Larvae Fed Palm Fruit Juice (PFJ) Delay Pupation via Expression Regulation of Hormetic Stress Response Genes Linked to Ageing and Longevity. Exp. Gerontol..

[71] Wang C., Wheeler C.T., Alberico T., Sun X., Seeberger J., Laslo M., Spangler E., Kern B., de Cabo R., Zou S. (2013). The Effect of Resveratrol on Lifespan Depends on Both Gender and Dietary Nutrient Composition in *Drosophila melanogaster*. Age.

[72] Hewlings S.J., Draayer K., Kalman D.S. (2021). Palm Fruit Bioactive Complex (PFBc), a Source of Polyphenols, Demonstrates Potential Benefits for Inflammaging and Related Cognitive Function. Nutrients.

[73] Syarifah-Noratiqah S.-B., Zulfarina M.S., Ahmad S.U., Fairus S., Naina-Mohamed I. (2019). The Pharmacological Potential of Oil Palm Phenolics (OPP) Individual Components. Int. J. Med. Sci..

[74] Bolsinger J., Pronczuk A., Sambanthamurthi R., Hayes K.C. (2014). Anti-Diabetic Effects of Palm Fruit Juice in the Nile Rat (*Arvicanthis niloticus*). J. Nutr. Sci..

[75] Chattopadhyay D., Sen S., Chatterjee R., Roy D., James J., Thirumurugan K. (2016). Context- and Dose-Dependent Modulatory Effects of Naringenin on Survival and Development of *Drosophila melanogaster*. Biogerontology.

[76] Hernández-Aquino E., Muriel P. (2018). Beneficial Effects of Naringenin in Liver Diseases: Molecular Mechanisms. World J. Gastroenterol..

[77] Wang Q., Ou Y., Hu G., Wen C., Yue S., Chen C., Xu L., Xie J., Dai H., Xiao H. (2020). Naringenin Attenuates Non-Alcoholic Fatty Liver Disease by down-Regulating the NLRP3/NF-ΚB Pathway in Mice. Br. J. Pharmacol..

[78] Lushchak O.V., Gospodaryov D.V., Rovenko B.M., Yurkevych I.S., Perkhulyn N.V., Lushchak V.I. (2014). Specific Dietary Carbohydrates Differentially Influence the Life Span and Fecundity of *Drosophila melanogaster*. J. Gerontol. A Biol. Sci. Med. Sci..

[79] Lushchak O.V., Gospodaryov D.V., Rovenko B.M., Glovyak A.D., Yurkevych I.S., Klyuba V.P., Shcherbij M.V., Lushchak V.I. (2012). Balance between Macronutrients Affects Life Span and Functional Senescence in Fruit Fly *Drosophila melanogaster*. J. Gerontol. A Biol. Sci. Med. Sci..

[80] LeMone P. (1999). Vitamins and Minerals. J. Obstet. Gynecol. Neonatal Nurs..

[81] Capozzi A., Scambia G., Lello S. (2020). Calcium, Vitamin D, Vitamin K2, and Magnesium Supplementation and Skeletal Health. Maturitas.

[82] Granger M., Eck P. (2018). Dietary Vitamin C in Human Health. Adv. Food Nutr. Res..

[83] Smith A.D., Warren M.J., Refsum H. (2018). Vitamin B12. Adv. Food Nutr. Res..

[84] Contestabile R., di Salvo M.L., Bunik V., Tramonti A., Vernì F. (2020). The Multifaceted Role of Vitamin B6 in Cancer: Drosophila as a Model System to Investigate DNA Damage. Open Biol..

[85] Ernst I.M.A., Pallauf K., Bendall J.K., Paulsen L., Nikolai S., Huebbe P., Roeder T., Rimbach G. (2013). Vitamin E Supplementation and Lifespan in Model Organisms. Ageing Res. Rev..

[86] Gnocchini E., Pilesi E., Schiano L., Vernì F. (2022). Vitamin B6 Deficiency Promotes Loss of Heterozygosity (LOH) at the Drosophila&Nbsp;Warts (Wts) Locus. Int. J. Mol. Sci..

[87] Merigliano C., Mascolo E., La Torre M., Saggio I., Vernì F. (2018). Protective Role of Vitamin B6 (PLP) against DNA Damage in Drosophila Models of Type 2 Diabetes. Sci. Rep..

[88] Nan Y., Lin J., Cui Y., Yao J., Yang Y., Li Q. (2021). Protective Role of Vitamin B6 against Mitochondria Damage in Drosophila Models of SCA3. Neurochem. Int..

[89] Green M.R., Sambrook J. (2021). Biotin. Cold Spring Harb. Protoc..

[90] Landenberger A., Kabil H., Harshman L.G., Zempleni J. (2004). Biotin Deficiency Decreases Life Span and Fertility but Increases Stress Resistance in *Drosophila melanogaster*. J. Nutr. Biochem..

[91] León-Del-Río A. (2019). Biotin in Metabolism, Gene Expression, and Human Disease. J. Inherit. Metab. Dis..

[92] Mock D.M. (2017). Biotin: From Nutrition to Therapeutics. J. Nutr..

[93] Wongkittichote P., Mew N.A., Chapman K.A. (2017). Propionyl-CoA Carboxylase—A Review. Mol. Genet. Metab..

[94] Mathers J.C. (2017). Nutrigenomics in the Modern Era. Proc. Nutr. Soc..

[95] Peña-Romero A.C., Navas-Carrillo D., Marín F., Orenes-Piñero E. (2018). The Future of Nutrition: Nutrigenomics and Nutrigenetics in Obesity and Cardiovascular Diseases. Crit. Rev. Food. Sci. Nutr..

[96] Ye J., Cui X., Loraine A., Bynum K., Kim N.C., White G., De Luca M., Garfinkel M.D., Lu X., Ruden D.M. (2007). Methods for Nutrigenomics and Longevity Studies in Drosophila: Effects of Diets High in Sucrose, Palmitic Acid, Soy, or Beef. Methods Mol. Biol..

[97] Ming Q.-L., Cheng C. (2012). Influence of Nutrition on Male Development and Reproduction in Tribolium Castaneum. J. Econ. Entomol..

[98] Grünwald S., Adam I.V., Gurmai A.-M., Bauer L., Boll M., Wenzel U. (2013). The Red Flour Beetle Tribolium Castaneum as a Model to Monitor Food Safety and Functionality. Adv. Biochem. Eng. Biotechnol..

[99] Schröder R., Beermann A., Wittkopp N., Lutz R. (2008). From Development to Biodiversity--Tribolium Castaneum, an Insect Model Organism for Short Germband Development. Dev. Genes Evol..

[100] Richards S., Gibbs R.A., Weinstock G.M., Brown S.J., Denell R., Beeman R.W., Gibbs R., Beeman R.W., Brown S.J., Tribolium Genome Sequencing Consortium (2008). The Genome of the Model Beetle and Pest Tribolium Castaneum. Nature.

[101] Grünwald S., Stellzig J., Adam I.V., Weber K., Binger S., Boll M., Knorr E., Twyman R.M., Vilcinskas A., Wenzel U. (2013). Longevity in the Red Flour Beetle Tribolium Castaneum Is Enhanced by Broccoli and Depends on Nrf-2, Jnk-1 and Foxo-1 Homologous Genes. Genes Nutr..

[102] Cheng S.-Q., Xia Y.-Y., He J.-L., Liu X.-Q., Chen X.-M., Ding Y.-B., Wang Y.-X., Peng B., Tu B.-J. (2013). Neurotoxic Effect of Subacute Benzo(a)Pyrene Exposure on Gene and Protein Expression in Sprague-Dawley Rats. Environ. Toxicol. Pharmacol..

[103] Ming Q.-L., Lewis S.M. (2010). Pheromone Production by Male Tribolium Castaneum (Coleoptera: Tenebrionidae) Is Influenced by Diet Quality. J. Econ. Entomol..

[104] Yadav M.K., Kumari I., Singh B., Sharma K.K., Tiwari S.K. (2022). Probiotics, Prebiotics and Synbiotics: Safe Options for next-Generation Therapeutics. Appl. Microbiol. Biotechnol..

[105] Żółkiewicz J., Marzec A., Ruszczyński M., Feleszko W. (2020). Postbiotics-A Step Beyond Pre- and Probiotics. Nutrients.

[106] Grau T., Vilcinskas A., Joop G. (2017). Probiotic Enterococcus Mundtii Isolate Protects the Model Insect Tribolium Castaneum against Bacillus Thuringiensis. Front. Microbiol..

[107] Piras C., Roncada P., Rodrigues P.M., Bonizzi L., Soggiu A. (2016). Proteomics in Food: Quality, Safety, Microbes, and Allergens. Proteomics.

[108] Jorjão A.L., Oliveira L.D., Scorzoni L., Figueiredo-Godoi L.M.A., Prata M.C.A., Jorge A.O.C., Junqueira J.C. (2018). From Moths to Caterpillars: Ideal Conditions for *Galleria mellonella* Rearing for in Vivo Microbiological Studies. Virulence.

[109] Kwadha C.A., Ong’amo G.O., Ndegwa P.N., Raina S.K., Fombong A.T. (2017). The Biology and Control of the Greater Wax Moth, *Galleria mellonella*. Insects.

[110] Firacative C., Khan A., Duan S., Ferreira-Paim K., Leemon D., Meyer W. (2020). Rearing and Maintenance of *Galleria mellonella* and Its Application to Study Fungal Virulence. J. Fungi.

[111] Cé R., Silva R.C., Trentin D.S., Marchi J.G.B.D., Paese K., Guterres S.S., Macedo A.J., Pohlmann A.R. (2020). *Galleria mellonella* Larvae as an In Vivo Model to Evaluate the Toxicity of Polymeric Nanocapsules. J. Nanosci. Nanotechnol..

[112] Junqueira J.C., Mylonakis E., Borghi E. (2021). *Galleria mellonella* Experimental Model: Advances and Future Directions. Pathog. Dis..

[113] Ménard G., Rouillon A., Cattoir V., Donnio P.-Y. (2021). *Galleria mellonella* as a Suitable Model of Bacterial Infection: Past, Present and Future. Front. Cell. Infect. Microbiol..

[114] Singkum P., Suwanmanee S., Pumeesat P., Luplertlop N. (2019). A Powerful in Vivo Alternative Model in Scientific Research: *Galleria mellonella*. Acta Microbiol. Immunol. Hung..

[115] Smith D.F.Q., Casadevall A. (2021). Fungal Immunity and Pathogenesis in Mammals versus the Invertebrate Model Organism *Galleria mellonella*. Pathog. Dis..

[116] Tao Y., Duma L., Rossez Y. (2021). *Galleria mellonella* as a Good Model to Study Acinetobacter Baumannii Pathogenesis. Pathogens.

[117] Lange A., Beier S., Huson D.H., Parusel R., Iglauer F., Frick J.-S. (2018). Genome Sequence of *Galleria mellonella* (Greater Wax Moth). Genome Announc..

[118] Reddy V.S., Palika R., Ismail A., Pullakhandam R., Reddy G.B. (2018). Nutrigenomics: Opportunities & Challenges for Public Health Nutrition. Indian J. Med. Res..

[119] Kangassalo K., Valtonen T.M., Sorvari J., Kecko S., Pölkki M., Krams I., Krama T., Rantala M.J. (2018). Independent and Interactive Effects of Immune Activation and Larval Diet on Adult Immune Function, Growth and Development in the Greater Wax Moth (*Galleria mellonella*). J. Evol. Biol..

[120] Krams I., Kecko S., Kangassalo K., Moore F.R., Jankevics E., Inashkina I., Krama T., Lietuvietis V., Meija L., Rantala M.J. (2015). Effects of Food Quality on Trade-Offs among Growth, Immunity and Survival in the Greater Wax Moth *Galleria mellonella*. Insect Sci..

[121] Banville N., Browne N., Kavanagh K. (2012). Effect of Nutrient Deprivation on the Susceptibility of *Galleria mellonella* Larvae to Infection. Virulence.

[122] Dimofski P., Meyre D., Dreumont N., Leininger-Muller B. (2021). Consequences of Paternal Nutrition on Offspring Health and Disease. Nutrients.

[123] Rossoni R.D., Fuchs B.B., de Barros P.P., Velloso M.D.S., Jorge A.O.C., Junqueira J.C., Mylonakis E. (2017). Lactobacillus Paracasei Modulates the Immune System of *Galleria mellonella* and Protects against Candida Albicans Infection. PLoS ONE.

[124] Scalfaro C., Iacobino A., Nardis C., Franciosa G. (2017). *Galleria mellonella* as an in vivo model for assessing the protective activity of probiotics against gastrointestinal bacterial pathogens. FEMS Microbiol. Lett..

[125] Wollowski I., Rechkemmer G., Pool-Zobel B.L. (2001). Protective Role of Probiotics and Prebiotics in Colon Cancer. Am. J. Clin. Nutr..

[126] Grounta A., Harizanis P., Mylonakis E., Nychas G.-J.E., Panagou E.Z. (2016). Investigating the Effect of Different Treatments with Lactic Acid Bacteria on the Fate of Listeria Monocytogenes and Staphylococcus Aureus Infection in *Galleria mellonella* Larvae. PLoS ONE.

[127] Jorjão A.L., de Oliveira F.E., Leão M.V.P., Jorge A.O.C., de Oliveira L.D. (2018). Effect of Lactobacillus Rhamnosus on the Response of *Galleria mellonella* against Staphylococcus Aureus and Escherichia Coli Infections. Arch. Microbiol..

[128] Ribeiro F.C., de Barros P.P., Rossoni R.D., Junqueira J.C., Jorge A.O.C. (2017). Lactobacillus Rhamnosus Inhibits Candida Albicans Virulence Factors in Vitro and Modulates Immune System in *Galleria mellonella*. J. Appl. Microbiol..

[129] Rossoni R.D., Dos Santos Velloso M., Figueiredo L.M.A., Martins C.P., Jorge A.O.C., Junqueira J.C. (2018). Clinical Strains of Lactobacillus Reduce the Filamentation of Candida Albicans and Protect *Galleria mellonella* against Experimental Candidiasis. Folia Microbiol..

[130] Vilela S.F.G., Barbosa J.O., Rossoni R.D., Santos J.D., Prata M.C.A., Anbinder A.L., Jorge A.O.C., Junqueira J.C. (2015). Lactobacillus Acidophilus ATCC 4356 Inhibits Biofilm Formation by C. Albicans and Attenuates the Experimental Candidiasis in *Galleria mellonella*. Virulence.

[131] Dhinaut J., Balourdet A., Teixeira M., Chogne M., Moret Y. (2017). A Dietary Carotenoid Reduces Immunopathology and Enhances Longevity through an Immune Depressive Effect in an Insect Model. Sci. Rep..

[132] Chang M.X., Xiong F. (2020). Astaxanthin and Its Effects in Inflammatory Responses and Inflammation-Associated Diseases: Recent Advances and Future Directions. Molecules.

[133] Higuera-Ciapara I., Félix-Valenzuela L., Goycoolea F.M. (2006). Astaxanthin: A Review of Its Chemistry and Applications. Crit Rev Food. Sci. Nutr..

[134] Kishimoto Y., Yoshida H., Kondo K. (2016). Potential Anti-Atherosclerotic Properties of Astaxanthin. Mar. Drugs.

[135] Sztretye M., Dienes B., Gönczi M., Czirják T., Csernoch L., Dux L., Szentesi P., Keller-Pintér A. (2019). Astaxanthin: A Potential Mitochondrial-Targeted Antioxidant Treatment in Diseases and with Aging. Oxid. Med. Cell. Longev..

[136] Davies S., Kattel R., Bhatia B., Petherwick A., Chapman T. (2005). The Effect of Diet, Sex and Mating Status on Longevity in Mediterranean Fruit Flies (Ceratitis Capitata), Diptera: Tephritidae. Exp. Gerontol..

[137] Chen E.-H., Wei D., Wei D.-D., Yuan G.-R., Wang J.-J. (2013). The Effect of Dietary Restriction on Longevity, Fecundity, and Antioxidant Responses in the Oriental Fruit Fly, Bactrocera Dorsalis (Hendel) (Diptera: Tephritidae). J. Insect Physiol..

[138] Duregon E., Pomatto-Watson L.C.D.D., Bernier M., Price N.L., de Cabo R. (2021). Intermittent Fasting: From Calories to Time Restriction. GeroScience.

[139] Fontana L. (2009). The Scientific Basis of Caloric Restriction Leading to Longer Life. Curr. Opin. Gastroenterol..

[140] Fanson B.G., Weldon C.W., Pérez-Staples D., Simpson S.J., Taylor P.W. (2009). Nutrients, Not Caloric Restriction, Extend Lifespan in Queensland Fruit Flies (*Bactrocera tryoni*). Aging Cell.

[141] Schatral A. (1993). Diet Influences Male-Female Interactions in the BushcricketRequena Verticalis (Orthoptera: Tettigoniidae). J. Insect Behav..

[142] Droney D.C. (1998). The Influence of the Nutritional Content of the Adult Male Diet on Testis Mass, Body Condition and Courtship Vigour in a Hawaiian Drosophila. Funct. Ecol..

[143] Stoffolano J.G., Tobin E.N., Wilson J., Yin C.-M. (1995). Diet Affects Insemination and Sexual Activity in Male Phormia Regina (Diptera: Calliphoridae). Ann. Entomol. Soc. Am..

[144] Engqvist L., Sauer K.P. (2003). Influence of Nutrition on Courtship and Mating in the Scorpionfly Panorpa Cognata (Mecoptera, Insecta). Ethology.

[145] Angel T., Aryal U. (2020). Impact of Gut Microbiota on Host by Exploring Proteomics. Gut Microbiome and Its Impact on Health and Diseases.

[146] Arias-Rojas A., Iatsenko I. (2022). The Role of Microbiota in *Drosophila melanogaster* Aging. Front. Aging.

[147] Gérard C., Vidal H. (2019). Impact of Gut Microbiota on Host Glycemic Control. Front. Endocrinol..

[148] Gould A.L., Zhang V., Lamberti L., Jones E.W., Obadia B., Korasidis N., Gavryushkin A., Carlson J.M., Beerenwinkel N., Ludington W.B. (2018). Microbiome Interactions Shape Host Fitness. Proc. Natl. Acad. Sci. USA.

[149] Lee H.-Y., Lee S.-H., Lee J.-H., Lee W.-J., Min K.-J. (2019). The Role of Commensal Microbes in the Lifespan of *Drosophila melanogaster*. Aging.

[150] Martin A.M., Sun E.W., Rogers G.B., Keating D.J. (2019). The Influence of the Gut Microbiome on Host Metabolism Through the Regulation of Gut Hormone Release. Front. Physiol..

[151] Fan X., Gaur U., Yang M. (2018). Intestinal Homeostasis and Longevity: Drosophila Gut Feeling. Adv. Exp. Med. Biol..

[152] Kim S., Jazwinski S.M. (2018). The Gut Microbiota and Healthy Aging: A Mini-Review. Gerontology.

[153] Chiang M.-H., Ho S.-M., Wu H.-Y., Lin Y.-C., Tsai W.-H., Wu T., Lai C.-H., Wu C.-L. (2022). Drosophila Model for Studying Gut Microbiota in Behaviors and Neurodegenerative Diseases. Biomedicines.

[154] Royet J. (2011). Epithelial Homeostasis and the Underlying Molecular Mechanisms in the Gut of the Insect Model *Drosophila melanogaster*. Cell. Mol. Life Sci..

[155] Kitani-Morii F., Friedland R.P., Yoshida H., Mizuno T. (2021). Drosophila as a Model for Microbiota Studies of Neurodegeneration. J. Alzheimers Dis..

[156] Kong Y., Wang L., Jiang B. (2021). The Role of Gut Microbiota in Aging and Aging Related Neurodegenerative Disorders: Insights from Drosophila Model. Life.

[157] Baenas N., Wagner A.E. (2019). *Drosophila melanogaster* as an Alternative Model Organism in Nutrigenomics. Genes Nutr..

[158] Erkosar B., Leulier F. (2014). Transient Adult Microbiota, Gut Homeostasis and Longevity: Novel Insights from the Drosophila Model. FEBS Lett..

[159] Lesperance D.N.A., Broderick N.A. (2020). Gut Bacteria Mediate Nutrient Availability in Drosophila Diets. Appl. Environ. Microbiol..

[160] Clark R.I., Walker D.W. (2018). Role of Gut Microbiota in Aging-Related Health Decline: Insights from Invertebrate Models. Cell. Mol. Life Sci..

[161] Maynard C., Weinkove D. (2018). The Gut Microbiota and Ageing. Subcell. Biochem..

[162] Aarsland D., Creese B., Politis M., Chaudhuri K.R., Ffytche D.H., Weintraub D., Ballard C. (2017). Cognitive Decline in Parkinson Disease. Nat. Rev. Neurol..

[163] Jellinger K.A. (2018). Dementia with Lewy Bodies and Parkinson’s Disease-Dementia: Current Concepts and Controversies. J. Neural Transm..

[164] Soria Lopez J.A., González H.M., Léger G.C. (2019). Alzheimer’s Disease. Handb. Clin. Neurol..

[165] Weller J., Budson A. (2018). Current Understanding of Alzheimer’s Disease Diagnosis and Treatment. F1000Research.

[166] Tan F.H.P., Liu G., Lau S.-Y.A., Jaafar M.H., Park Y.-H., Azzam G., Li Y., Liong M.-T. (2020). Lactobacillus Probiotics Improved the Gut Microbiota Profile of a *Drosophila melanogaster* Alzheimer’s Disease Model and Alleviated Neurodegeneration in the Eye. Benef. Microbes.

[167] Liu G., Tan F.H.-P., Lau S.-Y.A., Jaafar M.H., Chung F.Y.-L., Azzam G., Liong M.-T., Li Y. (2022). Lactic Acid Bacteria Feeding Reversed the Malformed Eye Structures and Ameliorated Gut Microbiota Profiles of *Drosophila melanogaster* Alzheimer’s Disease Model. J. Appl. Microbiol..

[168] Kim S.-H., Lee W.-J. (2014). Role of DUOX in Gut Inflammation: Lessons from Drosophila Model of Gut-Microbiota Interactions. Front. Cell. Infect. Microbiol..

[169] Charroux B., Royet J. (2012). Gut-Microbiota Interactions in Non-Mammals: What Can We Learn from Drosophila?. Semin. Immunol..

[170] Agarwal A., Agashe D. (2020). The Red Flour Beetle Tribolium Castaneum: A Model for Host-Microbiome Interactions. PLoS ONE.

[171] Wang X., Zhong Z., Chen X., Hong Z., Lin W., Mu X., Hu X., Zheng H. (2021). High-Fat Diets with Differential Fatty Acids Induce Obesity and Perturb Gut Microbiota in Honey Bee. Int. J. Mol. Sci..

[172] Allonsius C.N., Van Beeck W., De Boeck I., Wittouck S., Lebeer S. (2019). The Microbiome of the Invertebrate Model Host *Galleria mellonella* Is Dominated by Enterococcus. Anim. Microbiome.

[173] Carocho M., Morales P., Ferreira I.C.F.R. (2017). Sweeteners as Food Additives in the XXI Century: A Review of What Is Known, and What Is to Come. Food. Chem. Toxicol..

[174] Choi J.R., Yong K.W., Choi J.Y., Cowie A.C. (2019). Emerging Point-of-Care Technologies for Food Safety Analysis. Sensors.

[175] Trasande L., Shaffer R.M., Sathyanarayana S., COUNCIL ON ENVIRONMENTAL HEALTH (2018). Food Additives and Child Health. Pediatrics.

[176] Stadler R.H., Alfred S., Gökmen V. (2015). Acrylamide formation mechanisms. Acrylamide in Food Analysis, Content and Potential Health Effects.

[177] Michalak J., Czarnowska-Kujawska M., Klepacka J., Gujska E. (2020). Effect of Microwave Heating on the Acrylamide Formation in Foods. Molecules.

[178] Asadi S., Aalami M., Shoeibi S., Kashaninejad M., Ghorbani M., Delavar M. (2020). Effects of Different Roasting Methods on Formation of Acrylamide in Pistachio. Food Sci. Nutr..

[179] Bušová M., Bencko V., Kromerová K., Nadjo I., Babjaková J. (2020). Occurrence of Acrylamide in Selected Food Products. Cent. Eur. J. Public Health.

[180] Grünwald S., Niedermeier J., Wenzel U. (2015). Hormesis Is Induced in the Red Flour Beetle Tribolium Castaneum through Ingestion of Charred Toast. Eur. J. Nutr..

[181] Knight M., McWilliam S., Peck S., Koutsidis G., Chope G., Puddephat I., Wedzicha B. (2021). Kinetic Modelling of Acrylamide Formation during the Frying of Potato Chips. Food Chem..

[182] Rifai L., Saleh F.A. (2020). A Review on Acrylamide in Food: Occurrence, Toxicity, and Mitigation Strategies. Int. J. Toxicol..

[183] Singh L., Varshney J.G., Agarwal T. (2016). Polycyclic Aromatic Hydrocarbons’ Formation and Occurrence in Processed Food. Food Chem..

[184] EU European Commission Regulation of 20 November 2017 Establishing Mitigation Measures and Benchmark Levels for the Reduction of the Presence of Acrylamide in Food (2017/2158). https://eur-lex.europa.eu/eli/reg/2017/2158/oj.

[185] EU European Commission Recommendation of 7 November 2019 on the Monitoring of the Presence of Acrylamide in Certain Foods (2019/1888/EU). Commission Recommendation. https://eur-lex.europa.eu/legal-content/EN/TXT/?uri=CELEX%3A32019H1888.

[186] Grünwald S., Gurmai A.-M., Schuierer K., Boll M., Wenzel U. (2014). The Red Flour Beetle Tribolium Castaneum Allows for the Convenient Determination of Fitness and Survival as a Measure of Toxic Effects of the Food Contaminant Acrylamide. Food Addit. Contam. Part A Chem. Anal. Control Expo. Risk Assess..

[187] Fu L.-L., Zhao X.-Y., Ji L.-D., Xu J. (2019). Okadaic Acid (OA): Toxicity, Detection and Detoxification. Toxicon.

[188] Louzao M.C., Vieytes M.R., Botana L.M. (2005). Effect of Okadaic Acid on Glucose Regulation. Mini Rev. Med. Chem..

[189] Valdiglesias V., Prego-Faraldo M.V., Pásaro E., Méndez J., Laffon B. (2013). Okadaic Acid: More than a Diarrheic Toxin. Mar. Drugs.

[190] Coates C.J., Lim J., Harman K., Rowley A.F., Griffiths D.J., Emery H., Layton W. (2019). The Insect, *Galleria mellonella*, Is a Compatible Model for Evaluating the Toxicology of Okadaic Acid. Cell Biol. Toxicol..

[191] Maguire R., Duggan O., Kavanagh K. (2016). Evaluation of *Galleria mellonella* Larvae as an in Vivo Model for Assessing the Relative Toxicity of Food Preservative Agents. Cell Biol. Toxicol..

[192] van de Veerdonk F.L., Gresnigt M.S., Romani L., Netea M.G., Latgé J.-P. (2017). Aspergillus Fumigatus Morphology and Dynamic Host Interactions. Nat. Rev. Microbiol..

[193] Zhang Y., Wei W., Fan J., Jin C., Lu L., Fang W. (2020). Aspergillus Fumigatus Mitochondrial Acetyl Coenzyme A Acetyltransferase as an Antifungal Target. Appl. Environ. Microbiol..

[194] Paterson R.R.M., Lima N. (2017). Filamentous Fungal Human Pathogens from Food Emphasising Aspergillus, Fusarium and Mucor. Microorganisms.

[195] Durieux M.-F., Melloul É., Jemel S., Roisin L., Dardé M.-L., Guillot J., Dannaoui É., Botterel F. (2021). *Galleria mellonella* as a Screening Tool to Study Virulence Factors of Aspergillus Fumigatus. Virulence.

[196] Renwick J., Daly P., Reeves E.P., Kavanagh K. (2006). Susceptibility of Larvae of *Galleria mellonella* to Infection by Aspergillus Fumigatus Is Dependent upon Stage of Conidial Germination. Mycopathologia.

[197] Sheehan G., Clarke G., Kavanagh K. (2018). Characterisation of the Cellular and Proteomic Response of *Galleria mellonella* Larvae to the Development of Invasive Aspergillosis. BMC Microbiol..

[198] Fallon J.P., Reeves E.P., Kavanagh K. (2011). The Aspergillus Fumigatus Toxin Fumagillin Suppresses the Immune Response of *Galleria mellonella* Larvae by Inhibiting the Action of Haemocytes. Microbiology.

[199] Gorgus E., Hittinger M., Schrenk D. (2016). Estimates of Ethanol Exposure in Children from Food Not Labeled as Alcohol-Containing. J. Anal. Toxicol..

[200] Le Daré B., Lagente V., Gicquel T. (2019). Ethanol and Its Metabolites: Update on Toxicity, Benefits, and Focus on Immunomodulatory Effects. Drug Metab. Rev..

[201] Chandler J.A., Innocent L.V., Martinez D.J., Huang I.L., Yang J.L., Eisen M.B., Ludington W.B. (2022). Microbiome-by-Ethanol Interactions Impact *Drosophila melanogaster* Fitness, Physiology, and Behavior. iScience.

[202] Demir E., Demir F.T., Marcos R. (2022). Drosophila as a Suitable In Vivo Model in the Safety Assessment of Nanomaterials. Adv. Exp. Med. Biol..

[203] Chifiriuc M.C., Ratiu A.C., Popa M., Ecovoiu A.A. (2016). Drosophotoxicology: An Emerging Research Area for Assessing Nanoparticles Interaction with Living Organisms. Int. J. Mol. Sci..

[204] Pappus S.A., Mishra M. (2018). A Drosophila Model to Decipher the Toxicity of Nanoparticles Taken Through Oral Routes. Adv. Exp. Med. Biol..

[205] Alaraby M., Annangi B., Hernández A., Creus A., Marcos R. (2015). A Comprehensive Study of the Harmful Effects of ZnO Nanoparticles Using *Drosophila melanogaster* as an in Vivo Model. J. Hazard. Mater..

[206] Barik B.K., Mishra M. (2019). Nanoparticles as a Potential Teratogen: A Lesson Learnt from Fruit Fly. Nanotoxicology.

[207] Carmona E.R., Escobar B., Vales G., Marcos R. (2015). Genotoxic Testing of Titanium Dioxide Anatase Nanoparticles Using the Wing-Spot Test and the Comet Assay in Drosophila. Mutat. Res. Genet. Toxicol. Environ. Mutagen..

[208] Mishra M., Panda M. (2021). Reactive Oxygen Species: The Root Cause of Nanoparticle-Induced Toxicity in *Drosophila melanogaster*. Free Radic. Res..

[209] Yang P., Yang X., Sun L., Han X., Xu L., Gu W., Zhang M. (2022). Effects of Cadmium on Oxidative Stress and Cell Apoptosis in *Drosophila melanogaster* Larvae. Sci. Rep..

[210] Zhang Y., Wolosker M.B., Zhao Y., Ren H., Lemos B. (2020). Exposure to Microplastics Cause Gut Damage, Locomotor Dysfunction, Epigenetic Silencing, and Aggravate Cadmium (Cd) Toxicity in Drosophila. Sci. Total Environ..

[211] Sabat D., Patnaik A., Ekka B., Dash P., Mishra M. (2016). Investigation of Titania Nanoparticles on Behaviour and Mechanosensory Organ of *Drosophila melanogaster*. Physiol. Behav..

[212] Jovanović B., Cvetković V.J., Mitrović T.L. (2016). Effects of Human Food Grade Titanium Dioxide Nanoparticle Dietary Exposure on *Drosophila melanogaster* Survival, Fecundity, Pupation and Expression of Antioxidant Genes. Chemosphere.

